# Alterations in Tau Protein Level and Phosphorylation State in the Brain of the Autistic-Like Rats Induced by Prenatal Exposure to Valproic Acid

**DOI:** 10.3390/ijms22063209

**Published:** 2021-03-22

**Authors:** Magdalena Gąssowska-Dobrowolska, Agnieszka Kolasa-Wołosiuk, Magdalena Cieślik, Agnieszka Dominiak, Kristina Friedland, Agata Adamczyk

**Affiliations:** 1 Department of Cellular Signalling, Mossakowski Medical Research Institute, Polish Academy of Sciences, Pawińskiego 5, 02-106 Warsaw, Poland; mcieslik@imdik.pan.pl; 2 Department of Histology and Embryology, Pomeranian Medical University, Powstańców Wlkp. 72, 70-111 Szczecin, Poland; agnieszka.kolasa@pum.edu.pl; 3 Department of Biochemistry and Pharmacogenomics, Faculty of Pharmacy, Medical University of Warsaw, Żwirki i Wigury 61, 02-097 Warsaw, Poland; agnieszka.dominiak@wum.edu.pl; 4 Department of Pharmacology and Toxicology, Institute of Pharmaceutical and Biomedical Sciences, Johannes Gutenberg University, Staudingerweg 5, 55128 Mainz, Germany; kfriedla@uni-mainz.de

**Keywords:** α/β-tubulin, MAP-Tau, GSK-3β, CDK5, ERK1/2, Akt/mTOR signalling, valproic acid (VPA), autism spectrum disorders (ASD)

## Abstract

Autism spectrum disorder (ASD) is a neurodevelopmental condition characterized by deficient social interaction and communication besides repetitive, stereotyped behaviours. A characteristic feature of ASD is altered dendritic spine density and morphology associated with synaptic plasticity disturbances. Since microtubules (MTs) regulate dendritic spine morphology and play an important role in spine development and plasticity the aim of the present study was to investigate the alterations in the content of neuronal α/β-tubulin and Tau protein level as well as phosphorylation state in the valproic acid (VPA)-induced rat model of autism. Our results indicated that maternal exposure to VPA induces: (1) decrease the level of α/β-tubulin along with Tau accumulation in the hippocampus and cerebral cortex; (2) excessive Tau phosphorylation and activation of Tau-kinases: CDK5, ERK1/2, and p70S6K in the cerebral cortex; (3) up-regulation of mTOR kinase-dependent signalling in the hippocampus and cerebral cortex of adolescent rat offspring. Moreover, immunohistochemical staining showed histopathological changes in neurons (chromatolysis) in both analysed brain structures of rats prenatally exposed to VPA. The observed changes in Tau protein together with an excessive decrease in α/β-tubulin level may suggest destabilization and thus dysfunction of the MT cytoskeleton network, which in consequence may lead to the disturbance in synaptic plasticity and the development of autistic-like behaviours.

## 1. Introduction

Autism spectrum disorders (ASD) is a behaviourally defined heterogeneous complex of neurodevelopmental conditions characterized by persistent deficient social interaction, impaired communication as well as restricted or stereotyped patterns of behaviours, interests and activities, and abnormalities in sensory reactivity [1,2]. Epidemiological studies estimate that roughly 1% of the world’s population is thought to have an ASD, but exact numbers are highly dependent upon the still-evolving methodology [3,4,5]. A significant rise in the prevalence of the condition in recent years has increased the importance of clarifying its pathogenesis. 

While ASD shares characteristic features at the behavioural level, the pathogenesis of autism has not yet been clearly elucidated, and its underlying causes are highly heterogeneous. There may be many different agents that make a child more likely to have an ASD, including genetic, biological, and environmental factors as well as the interaction between them [6]. Exposure to maternal stressors, infectious agents, drugs, as well as environmental pollutants during the early phase of life may induce epigenetic changes, which have a latent and long-term impact on brain function, leading to various congenital malformations in offspring, and increase the risk of ASD [7,8].

Although the aetiology of ASD remains largely unknown, increasing evidence suggests that abnormal brain development, synaptic dysfunction, and disturbances in neuronal communication are involved in the pathogenesis of autism [9,10,11,12,13]. Studies of neurons from ASD patients as well as our previous results obtained from the valproic acid (VPA)-induced rat model of ASD revealed ultrastructural and molecular alterations in synapses [14] as well as morphological abnormalities within dendrites, axons, dendritic spines, and in the organization of the neural network [15,16,17,18,19], which may underlie the neuronal connectivity and neuroplasticity disturbances. 

The neuroplasticity mechanisms (including e.g.,: neurogenesis, axonal elongation, dendritic remodeling and retraction, neuronal branching, as well as synaptogenesis and synaptic turnover), which are dynamic processes that optimize the connectivity and function of neural circuits in response to a stimulus, require morphological and structural changes driven by rearrangement of the neuronal cytoskeletal components [12,17,20]. Cytoskeletal elements, including the microtubules (MTs) and microtubule-associated proteins (MAPs), are critical for the development and stabilization of axonal and dendritic processes, neuron production, and synaptic formation as well as neuronal communication [12,20,21,22,23]. A growing body of evidence suggests that many genes associated with a higher risk of ASD encode MT-associated proteins involved in cytoskeletal dynamics [12,22,23,24]. Genetic mutations or deregulated expression of MT-related genes suggest that the MT cytoskeleton is compromised in ASD, which can lead to detrimental developmental defects. 

MTs are one of the major structural elements of the cytoskeleton that consist of 13 polarized linear protofilaments associated laterally and formed by α- and β-tubulin heterodimers—MT building blocks [22,25]. MTs are highly dynamic intracellular polymers essential for neuronal growth, morphology, migration, and polarity [26]. In cooperation with several classes of binding proteins, MTs regulate long-distance intracellular cargo trafficking along axons and dendrites to establish appropriate neural connectivity [26]. The structure and dynamics of MTs are heavily dependent on the polymerisation/depolymerisation ratio and are adjusted by post-translational tubulin modifications as well as by different MT-interacting proteins [20,26]. One of the key proteins responsible for the stabilization and regulation of the MTs network is Tau (tubulin-associated unit), which is mainly found in the axonal compartment [27]. Therefore, Tau plays a particularly important role in the stabilization of axonal MTs thus enables axonal transport of signal molecules and trophic factors as well as participates in the processes of neurite growth and synaptogenesis [28,29]. The affinity of Tau for the MTs depends on its phosphorylation state [30,31]. In physiological conditions, Tau is in constant dynamic equilibrium, is briefly associated with MTs, then phosphorylated by kinases, which causes a short-term disconnection from MTs, while after dephosphorylation by phosphatases, it is reattached to MTs. Its excessive phosphorylation by Tau regulatory kinases such as glycogen synthase kinase-3β (GSK-3β), mitogen-activated protein kinase (MAP kinase), cyclin-dependent kinase 5 (CDK5), cAMP-dependent protein kinase A (PKA), protein kinase C (PKC), etc. allows MTs depolymerisation leading to destabilization of MTs and the neuronal cytoskeleton and consequently impairs mitochondrial respiration, axonal transport, post-synaptic function as well as compromises cell signalling, resulting in cognitive impairments [27,32,33,34,35,36,37]. Therefore, the proper functioning of Tau protein determines the maintenance of the correct structure of neurons and their synapses, as well as the proper course of neurotransmission and synaptic plasticity processes. Until now, changes in the level of Tau protein and the processes of its phosphorylation have been intensively studied in neurodegenerative diseases, in particular in Alzheimer’s disease (AD), in which hyperphosphorylated Tau protein is deposited in the brain in the form of neurofibrillary tangles (NFTs) and is involved in the molecular mechanisms of neuronal death [33,35,37,38,39]. Novel and most interesting data suggest that changes in the level of Tau protein and its phosphorylation state may also be important in the aetiology of neurodevelopmental disorders and in the formation of autistic-like behaviour [17,28,40,41,42,43,44]. However, there are relatively sparse data and the precise molecular mechanisms underlying the pathogenesis of autism are not fully elucidated. Although alterations in different MT-associated proteins have already been demonstrated in many neurodevelopmental disorders [12,22,23,25] as well as in ASD subjects and in various experimental models of ASD [12,17,22,23,45,46,47], no study has yet examined whether, in a rat model of environmentally triggered autism based on embryological exposure in utero to VPA, the fundamental MTs building block—α/β-tubulin and MAP-Tau protein are functionally altered. Investigation of the α/β-tubulin and MAP-Tau protein level in the VPA-induced ASD model is as yet lacking. Above all, the histological changes in the neurons after VPA exposure have not been investigated so far. Evaluation of the relationship between alterations in protein level and/or dysfunction of both α/β-tubulin and Tau and increased risk of ASD seems to be particularly important, considering that synaptic dysfunction is present in the pathogenesis of ASD and that MT structural proteins together with MT-interacting proteins are important regulators of synaptic formation and neuron production. 

Hence, the present work aimed to study alterations in the content of neuronal α/β-tubulin and Tau protein as well as the phosphorylation state of Tau in the VPA-induced rat model of ASD. Moreover, the involvement of a major Tau-kinases: GSK-3β, extracellular signal-regulated kinases 1 and 2 (ERK1/2), and CDK5 in VPA-evoked Tau phosphorylation were evaluated. Taking into account the commitment of the mechanistic target of rapamycin (mTOR) kinase to maintaining Tau protein homeostasis, we investigated also its activity status [44,48,49]. Additionally, we examined the structure of nerve cells. We studied the hippocampus and the cerebral cortex of young-adult male offspring as these brain areas that control many of the executive functions of the brain, including higher-order cognitive processes.

Our study for the first time showed a drastic decrease in the level of α/β-tubulin and significant accumulation of MAP-Tau protein along with histopathological changes seen in the cell body of neurons (chromatolysis) in both the hippocampus and cerebral cortex of adolescent offspring prenatally exposed to VPA. In addition, our results revealed that prenatal VPA administration caused excessive Tau phosphorylation in the cerebral cortex together with activation of ERK1/2, CDK5, and p70 ribosomal protein S6 (p70S6) kinases. All these alterations were accompanied by an increase in the activity of mTOR kinase in both analysed parts of the VPA-exposed offspring brain. In this study, we suggest that prenatal exposure to VPA by inducing brain region-dependent Tau phosphorylation may lead to a decreased capacity of Tau to assemble α/β-tubulin into MTs. Altogether, the observed changes in the cytoskeletal proteins along with pathologically altered neurons may suggest destabilization and thus dysfunction of the MT cytoskeleton network, which in consequence may lead to the disturbance of synaptic structure and connectivity, contributing to cognitive and behavioural abnormalities found in these animals [14]. Moreover, our results emphasised the importance of mTOR activation (in a brain region-specific manner) as a potential trigger of the molecular cascade, leading to Tau protein dyshomeostasis. 

## 2. Results

### 2.1. Prenatal Exposure to VPA Induced Changes in the Level of α/β-Tubulin and MAP-Tau 

**Figure 1:**
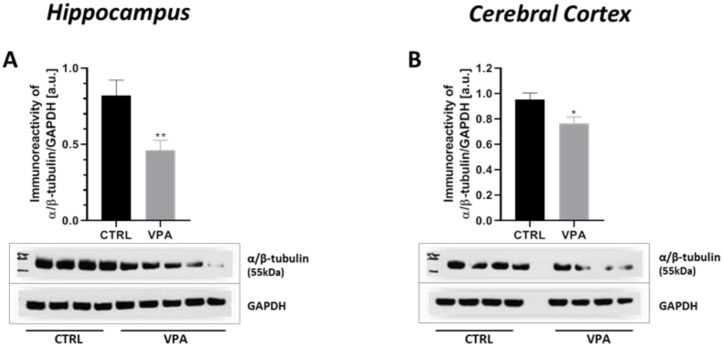
The effect of prenatal exposure to valproic acid (VPA) on the protein level of α/β-tubulin in the rat brain. Immunoreactivity of α/β-tubulin in control and VPA-exposed rats was monitored using Western blot analysis. Densitometric analysis and representative pictures of α/β-tubulin in the hippocampus (**A**) and cerebral cortex (**B**) were shown. Results were normalized to glyceraldehyde 3-phosphate dehydrogenase (GAPDH) levels. Data represent the mean values ± SEM from *n* = (11–14) independent experiments in the hippocampus: (**A**) *n* = 11 (CTRL), *n* = 14 (VPA); and *n* = (11) in the cerebral cortex: (**B**) *n* = 11 (CTRL), *n* = 11 (VPA). * *p* < 0.5, ** *p* < 0.01, vs. control.

**Figure 2:**
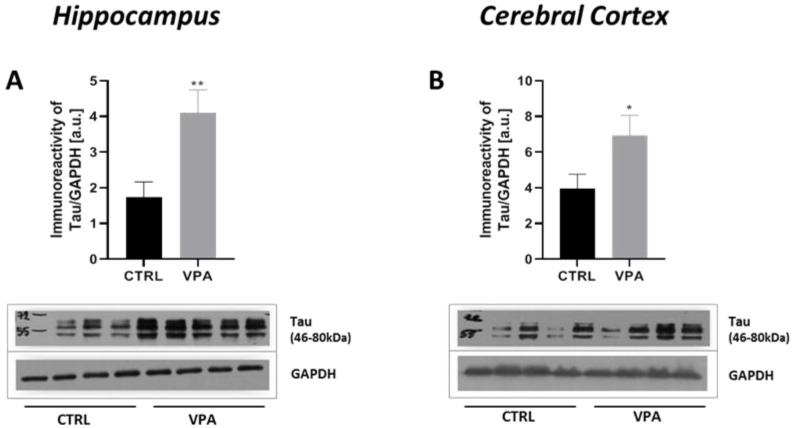
The effect of prenatal exposure to VPA on the protein level of total Tau in the rat brain. Immunoreactivity of total Tau in control and VPA-exposed rats was monitored using Western blot analysis. Densitometric analysis and representative pictures of total Tau in the hippocampus (**A**) and cerebral cortex (**B**) were shown. Results were normalized to GAPDH levels. Data represent the mean values ± SEM from *n* = (8) independent experiments in both the hippocampus and cerebral cortex: (**A**) *n* = 8 (CTRL), *n* = 8 (VPA); (**B**) *n* = 8 (CTRL), *n* = 8 (VPA). * *p* < 0.5, ** *p* < 0.01, vs. control.

**Figure 3:**
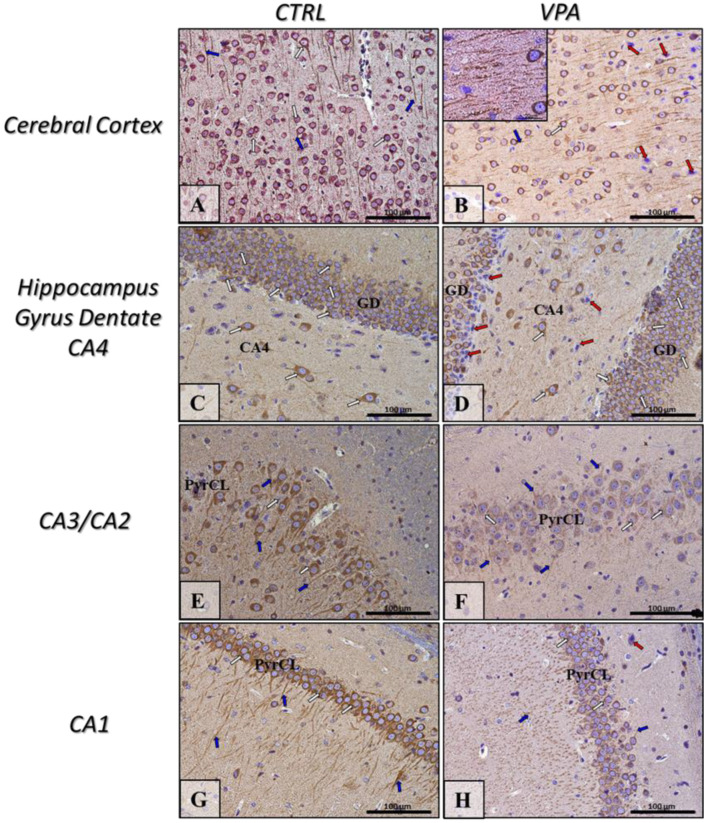
The effect of prenatal exposure to VPA on the immunoreactivity of α/β-tubulin in the rat brain. Representative microphotography showing immunoexpression of α/β-tubulin in the cerebral cortex and hippocampus proper (GD—Gyrus Dentate, CA1-CA4—the regions of Cornu Ammonis) of control rats (**A**,**C**,**E**,**G**) and VPA-treated rats (**B**,**D**,**F**,**H**). IHC reaction. Scale bar: 100 µm and 20 µm in the insertion of B (objective magnification ×40, ×100, respectively). α/β-tubulin-immunopositive perikaryon (white arrows) and their processes (blue arrows); red arrows—α/β-tubulin-immunonegative neurons with pathologic phenotype (chromatolysis—collapsed cell nucleus as a result of the negatively affected cytoskeletal network because of degradation of Nissl bodies and disturbances of cell metabolism); PyrCL—Pyramidal Cell Layer of CA1-CA3 regions. Representative pictures from *n* = 11 (CTRL) and *n* = 9 (VPA) independent experiments in both the hippocampus and cerebral cortex are presented.

**Figure 4:**
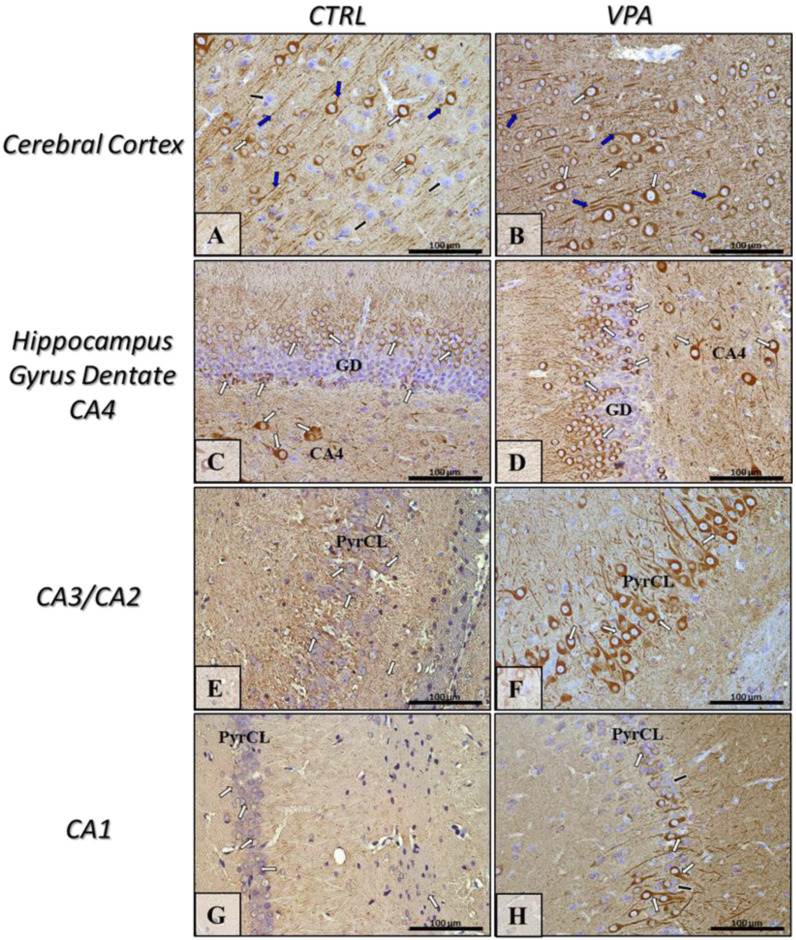
The effect of prenatal exposure to VPA on the immunoreactivity of total Tau protein in the rat brain. Representative microphotography showing immunoexpression of total Tau in the cerebral cortex and hippocampus proper (GD—Gyrus Dentate, CA1-CA4—the regions of Cornu Ammonis) of control rats (**A**,**C**,**E**,**G**) and VPA-treated rats (**B**,**D**,**F**,**H**). IHC reaction. Scale bar: 100 µm (objective magnification ×40). Tau-immunopositive perikaryons (white arrows) and their processes (blue arrows); black arrows—Tau-immunonegative cells; PyrCL—Pyramidal Cell Layer of CA1-CA3 regions. Representative pictures from *n* = 11 (CTRL) and *n* = 9 (VPA) independent experiments in both the hippocampus and cerebral cortex are presented.

To evaluate whether prenatal VPA exposure leads to changes in the expression of key proteins responsible for regulating MT assembly and stability, we measured the protein level of α/β-tubulin and MAP-Tau in both the hippocampus as well as the cerebral cortex of adolescent rats. The Western blot analysis indicated that exposure to VPA during embryonic development significantly decreased the level of α/β-tubulin protein by about 43% in the hippocampus (*p* = 0.0052) and by about 20% in the cerebral cortex (*p* = 0.0219) (Figure 1A,B). In turn, the analysis of the expression of Tau revealed a significant increase in this protein level in both the hippocampus (by about 136%, *p* = 0.0093) and cerebral cortex (by about 75%, *p* = 0.0479) of VPA offspring, compared to the control group (Figure 2A,B). The Western blot results were confirmed by immunohistochemical analysis, which revealed significantly lower immunoexpression of α/β-tubulin in the perikaryon of cortical nerve cells and their processes (white and blue arrows, respectively) in VPA rats (Figure 3B) than in control animals (Figure 3A). Moreover, in the cerebral cortex of VPA-exposed animals, we observed many α/β-tubulin immunonegative cells with the pathologically changed phenotype (Figure 3B, red arrows). In the control group, the expression of α/β-tubulin has a more compact pattern in processes of neurons, in opposite to VPA group, where along the nerve cell processes can observe vacuolization (Figure 3B, insertion). The same dependency we observed in the hippocampus. Neurons of Gyrus Dentate (GD) and CA4 region of VPA-exposed rats shown lower immunoexpression of α/β-tubulin (Figure 3D) and revealed many immunonegative cells with the pathologically changed phenotype (Figure 3D, red arrows) in opposite to control hippocampus (Figure 3C, white arrows). In the CA3/CA2 and CA1 regions of Cornu Ammonis of VPA-exposed rats, the expression of α/β-tubulin in the Pyramidal Cell Layer (PyrCL) (Figure 3F,H, white and blue arrows) was also lower than in the control animals (Figure 3E,G, white and blue arrows). The immunohistochemical analysis of Tau revealed more intensive expression of this protein in both the perikaryons of cortical neurons and their processes (white and blue arrows, respectively) of VPA-exposed animals, compared to control rats (Figure 4A,B). Similarly, in the hippocampus, the stronger immunoexpression of total Tau protein was visible in cells of the apical and basal area of Granular Cell Layer (GCL) of GD as well as in neurons of the CA4 region of VPA-treated animals (Figure 4D, white arrows) than in the control group (Figure 4C, white arrows). The immunoexpression of Tau protein in PyrCL of CA3/CA2 and CA1 regions of Cornu Ammonis was also higher in neurons of VPA animals (Figure 4F,H, white arrows) than in the control group (Figure 4E,G, white arrows). 

### 2.2. Prenatal Exposure to VPA Evoked Changes in the Phosphorylation Status of MAP-Tau Protein

**Figure 5:**
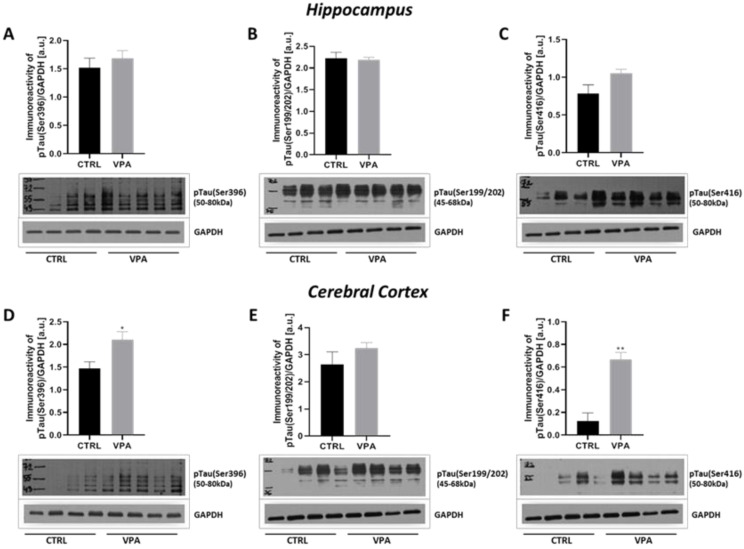
The effect of prenatal exposure to VPA on the phosphorylation state of Tau protein in the rat brain. Immunoreactivity of pTau(Ser396), pTau(Ser199/202), and pTau(Ser416) protein in control and VPA-exposed rats was monitored using Western blot analysis. Densitometric analysis and representative pictures of pTau(Ser396), pTau(Ser199/202), and pTau(Ser416) in the hippocampus (**A**–**C**) and cerebral cortex (**D**–**F**) were shown. Results were normalized to GAPDH levels. Data represent the mean values ± SEM from *n* = (4–8) independent experiments in the hippocampus: (**A**) *n* = 8 (CTRL), *n* = 8 (VPA); (**B**) *n* = 4 (CTRL), *n* = 4 (VPA); (**C**) *n* = 5 (CTRL), *n* = 5 (VPA); and *n* = (3–8) independent experiments in the cerebral cortex: (**D**) *n* = 8 (CTRL), *n* = 8 (VPA); (**E**) *n* = 4 (CTRL), *n* = 5 (VPA); (**F**) *n* = 3 (CTRL), *n* = 3 (VPA). * *p* < 0.5, ** *p* < 0.01, vs. control.

**Figure 6:**
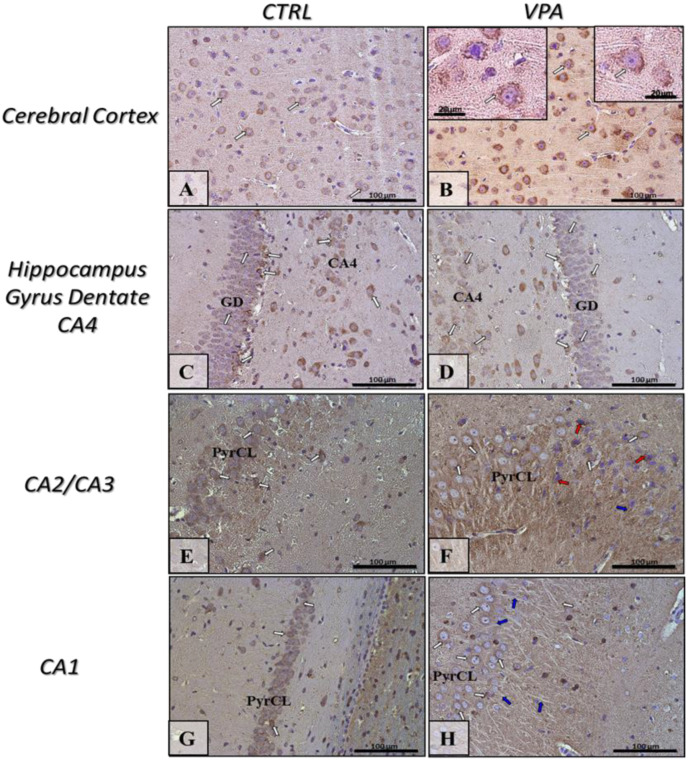
The effect of prenatal exposure to VPA on the immunoreactivity of Tau phosphorylated at (Ser396) in the rat brain. Representative microphotography showing immunoexpression of pTau(Ser396) in the cerebral cortex and hippocampus proper (GD—Gyrus Dentate, CA1-CA4—the regions of Cornu Ammonis) of control rats (**A**,**C**,**E**,**G**) and VPA-treated rats (**B**,**D**,**F**,**H**). IHC reaction. Scale bar: 100 µm and 20 µm in insertion of B (objective magnification ×40, ×100, respectively). pTau(Ser396)-immunopositive perikaryons (white arrows); blue arrows—the pTau(Ser396)-immunonegative nerve cells processes; red arrows—pTau(Ser396)-immunonegative neurons with pathologic phenotype (chromatolysis—collapsed cell nucleus as a result of negatively affected cytoskeletal network because of degradation of Nissl bodies and disturbances of cell metabolism); PyrCL—Pyramidal Cell Layer of CA1-CA3 regions. Representative pictures from *n* = 11 (CTRL) and *n* = 9 (VPA) independent experiments in both the hippocampus and cerebral cortex are presented.

**Figure 7:**
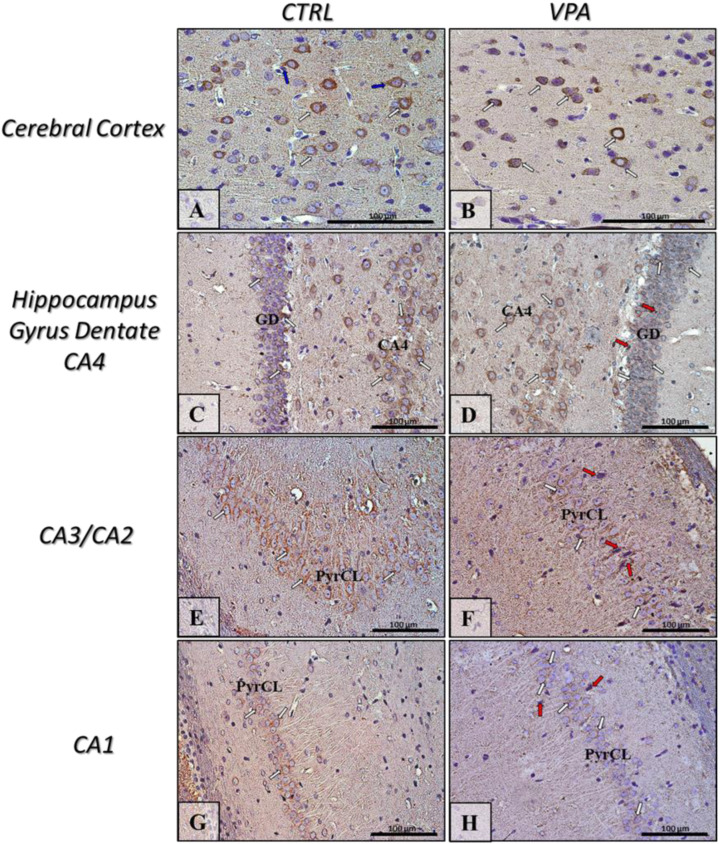
The effect of prenatal exposure to VPA on the immunoreactivity of Tau phosphorylated at (Ser199/202) in the rat brain. Representative microphotography showing immunoexpression of pTau(Ser199/202) in the cerebral cortex and hippocampus proper (GD—Gyrus Dentate, CA1-CA4—the regions of Cornu Ammonis) of control rats (**A**,**C**,**E**,**G**) and VPA-treated rats (**B**,**D**,**F**,**H**). IHC reaction. Scale bar: 100 µm (objective magnification ×40). pTau(Ser199/202)-immunopositive perikaryon (white arrows) and their processes (blue arrows); red arrows—pTau(Ser199/202)-immunonegative or -very low positive neurons with pathologic phenotype (chromatolysis); PyrCL—Pyramidal Cell Layer of CA1-CA3 regions. Representative pictures from *n* = 11 (CTRL) and *n* = 9 (VPA) independent experiments in both the hippocampus and cerebral cortex are presented.

**Figure 8:**
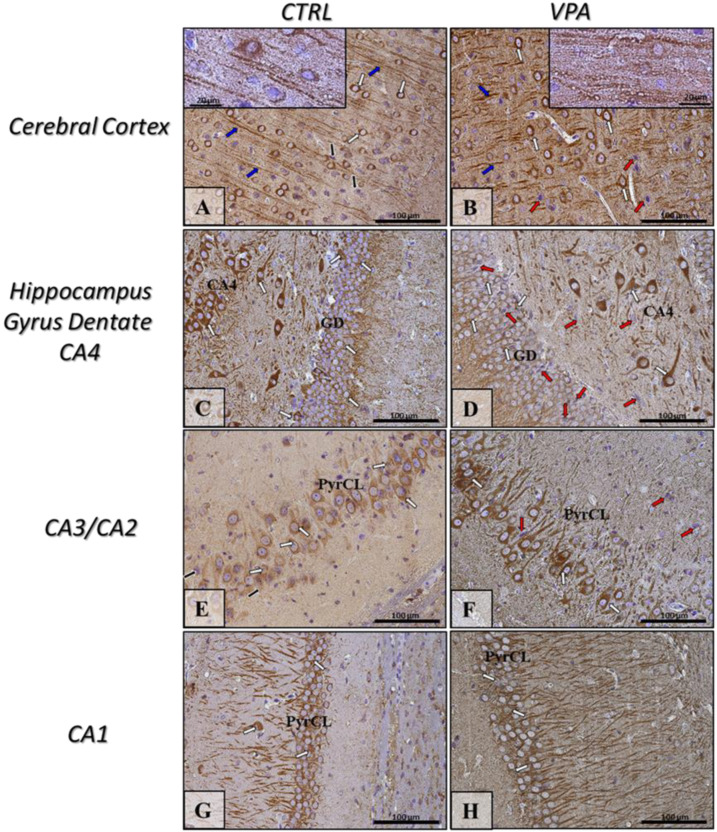
The effect of prenatal exposure to VPA on the immunoreactivity of Tau phosphorylated at (Ser416) in the rat brain. Representative microphotography showing immunoexpression of pTau(Ser416) in the cerebral cortex and hippocampus proper (GD—Gyrus Dentate, CA1-CA4—the regions of Cornu Ammonis) of control rats (**A**,**C**,**E**,**G**) and VPA-treated rats (**B**,**D**,**F**,**H**). IHC reaction. Scale bar: 100 µm and 20 µm in the insertion of A and B (objective magnification ×40, ×100, respectively). pTau(Ser416)-immunopositive perikaryon (white arrows) and their processes (blue arrows); red arrows—pTau(Ser416)-immunonegative neurons with pathologic phenotype (chromatolysis—collapsed cell nucleus as a result of the negatively affected cytoskeletal network because of degradation of Nissl bodies and disturbances of cell metabolism); PyrCL—Pyramidal Cell Layer of CA1-CA3 regions. Representative pictures from *n* = 11 (CTRL) and *n* = 9 (VPA) independent experiments in both the hippocampus and cerebral cortex are presented.

The function of Tau protein depends on its phosphorylation state. Abnormal hyperphosphorylation of Tau decreases its MT-binding capacity and disrupts MT stability. Therefore, we analysed the phosphorylation status of Tau at three different specific residues: (Ser396), (Ser199/202), and (Ser416). No changes in the level of phosphorylation of this protein were observed in the hippocampus of VPA animals, compared to control (Figure 5A–C). However, prenatal exposure to VPA significantly increased the level of pTau, phosphorylated at (Ser396) (by about 43%, *p* = 0.0111) and (Ser416) (by about 448%, *p* = 0.0048) in the cerebral cortex, compared to the respective control groups (Figure 5D,F) without effect on (Ser199/202) phosphorylation (Figure 5E). Western blot analysis was confirmed by the immunohistochemical investigation. The immunoexpression of pTau, phosphorylated at (Ser396) was apparently higher in the cortical perikaryon of VPA-exposed rats than in control (Figure 6A,B, white arrows) although with a more granulated/vacuolated pattern (Figure 6B, insertion—white arrows). Within hippocampus proper (GD and Cornu Ammonis), both the level and the pattern of pTau(Ser396) reactivity in both control and VPA groups was very comparable (Figure 6C–H, white arrows). In turn, the expression of pTau(Ser199/202) in the neurons of the cerebral cortex and all regions of the hippocampus in VPA-exposed rats was similar to the control group (Figure 7A–H, white arrows). However, in the VPA group there were some neurons in GD, CA4, and CA1–CA3 region of the hippocampus with pathologic phenotype (chromatolytic cells) almost without pTau(Ser199/202) immunoexpression (Figure 7D,F,H, red arrows). Moreover, the intensity of pTau(Ser416) expression in the neurons (cell bodies and their processes) in the cerebral cortex of the VPA-exposed group was a little bit higher than in control rats (Figure 8A,B; white and blue arrows), but in the VPA-treated rats, more neurons were pTau(Ser416)-immunonegative with a visible pathologically changed phenotype (Figure 8B, red arrow). Additionally, in the cerebral cortex of VPA rats the nerve cell processes (axons) were thicker, looks like distended, and along possess have many swollen vacuoles in opposite to control (Figure 8A,B, insertion). In turn, in all regions of the hippocampus (GD, CA4, CA3/CA2, and CA1) the immunoexpression of pTau(Ser416) was comparable in both analysed groups (Figure 8C–H, white arrows), but in VPA offspring were visible many immunonegative neurons with pathologic phenotype (Figure 8D,F, red arrows).

### 2.3. Prenatal Exposure to VPA Led to the Deregulation of Tau Kinases Activity in a Brain Region-Specific Manner

**Figure 9:**
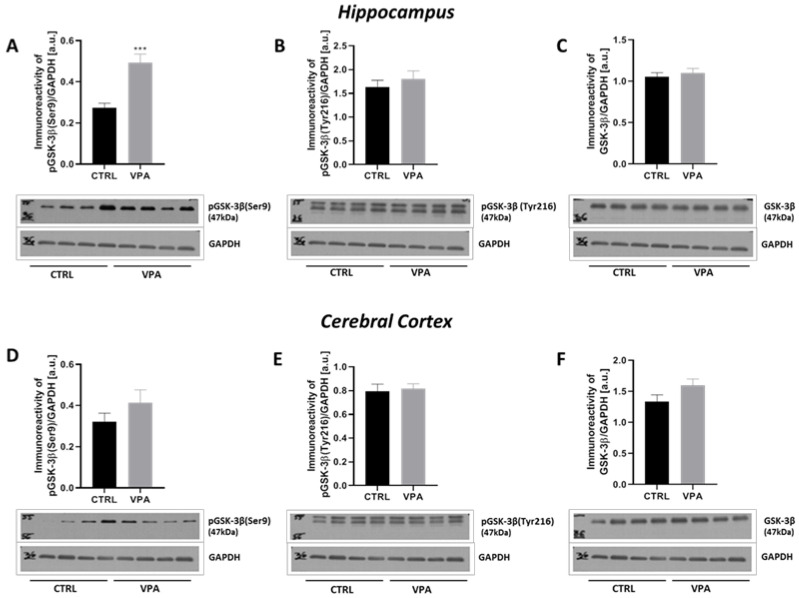
The effect of prenatal exposure to VPA on the GSK-3β in the rat brain. The phosphorylation status of GSK-3β at (Ser9) and (Tyr216) as well as the immunoreactivity of total GSK-3β in control and VPA-exposed rats were monitored using Western blot analysis. Densitometric analysis and representative pictures of pGSK-3β(Ser9), pGSK-3β(Tyr216) and total GSK-3β in the hippocampus (**A**–**C**) and cerebral cortex (**D**–**F**) were shown. Results were normalized to GAPDH levels. Data represent the means ± S.E.M. from *n* = (8–10) independent experiments in the hippocampus: (**A**) *n* = 8 (CTRL), *n* = 8 (VPA); (**B**) *n* = 10 (CTRL), *n* = 8 (VPA); (**C**) *n* = 10 (CTRL), *n* = 9 (VPA); and *n* = (7–9) in the cerebral cortex: (**D**) *n* = 7 (CTRL), *n* = 7 (VPA); (**E**) *n* = 9 (CTRL), *n* = 7 (VPA); (**F**) *n* = 9 (CTRL), *n* = 7 (VPA). *** *p* < 0.001, vs. control.

**Figure 10:**
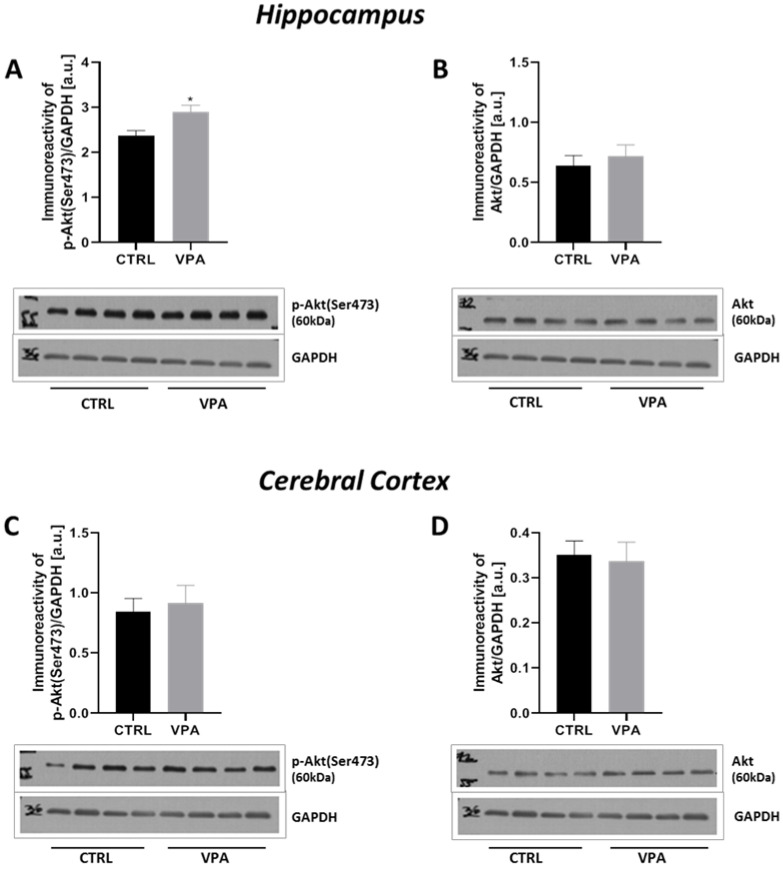
The effect of prenatal exposure to VPA on the Akt in the rat brain. Immunoreactivity of p-Akt(Ser473) and total Akt were determined using Western blot analysis. Densitometric analysis and representative pictures of p-Akt(Ser473) and total Akt in the hippocampus (**A**,**B**) and cerebral cortex (**C**,**D**) were shown. Results were normalized to GAPDH levels. Data represent the means ± S.E.M. from *n* = (8–9) independent experiments in the hippocampus: (**A**) *n* = 9 (CTRL), *n* = 8 (VPA); (**B**) *n* = 9 (CTRL), *n* = 8 (VPA); and *n* = (7–9) in the cerebral cortex: (**C**) *n* = 9 (CTRL), *n* = 8 (VPA); (**D**) *n* = 9 (CTRL), *n* = 7 (VPA). * *p* < 0.5, vs. control.

To evaluate the involvement of the major Tau kinases in VPA-induced Tau phosphorylation, the activity of GSK-3β, ERK1/2 and CDK5 was analysed. GSK-3β activity was evaluated by measurement of the phosphorylation status at both (Ser9) and (Tyr216). Moreover, its protein level was evaluated. As presented in Figure 9, prenatal exposure to VPA evoked a significant (*p* = 0.0004) increase in the level of p-GSK-3β(Ser9) by about 30% exclusively in the hippocampus, compared to the control group (Figure 9A,D). The immunoreactivity of p-GSK-3β(Tyr216) remained unchanged in both the hippocampus and cerebral cortex (Figure 9B,E), nor did protein levels (Figure 9C,F). The above data indicate inhibition of GSK-3β activity in the hippocampus of VPA offspring. To confirm the inhibitory effect of VPA on GSK-3β we analysed the activity of protein kinase B (Akt) kinase, a negative regulator of GSK-3β. The activity of Akt kinase was determined by measurement of its phosphorylation state at (Ser473). As shown in Figure 10A,C, the level of p-Akt(Ser473) was significantly (*p* = 0.0123) increased by about 23% only in the hippocampus of VPA-exposed rats, whereas the protein level of total Akt was unchanged in both analysed brain structure (Figure 10B,D). These results can be a confirmation the Akt-dependent inactivation of hippocampal GSK-3β kinase in response to VPA exposure during embryonic development. 

**Figure 11:**
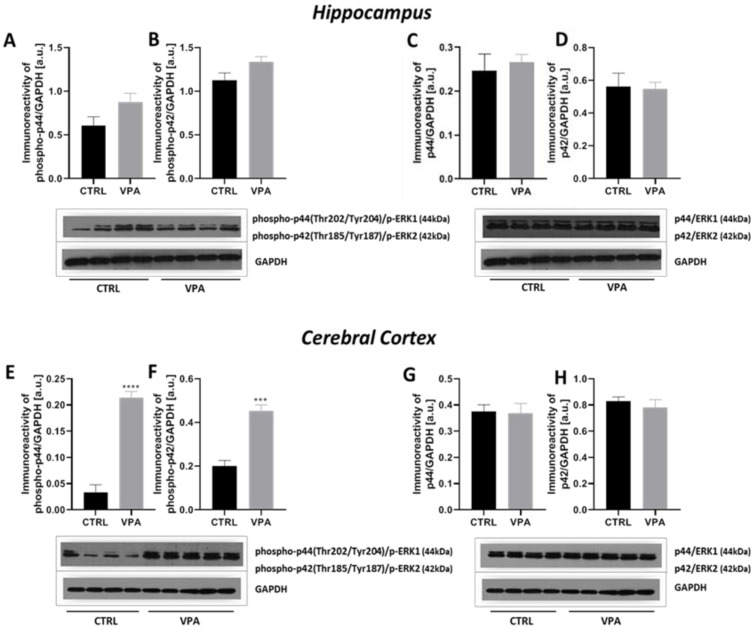
The effect of prenatal exposure to VPA on the p44/p42MAPK (ERK1/2) in the rat brain. Immunoreactivity of phospho-ERK1(Thr202/Tyr204), phospho-ERK2(Thr185/Tyr187), ERK1, and ERK2 were monitored using Western blot analysis. Densitometric analysis and representative pictures of phospho-ERK1, phospho-ERK2 as well as total ERK1 and ERK2 in the hippocampus (**A**–**D**) and cerebral cortex (**E**–**H**) were shown. Results were normalized to GAPDH levels. Data represent the means ± S.E.M. from *n* = (4–9) independent experiments in the hippocampus: (**A**) *n* = 9 (CTRL), *n* = 8 (VPA); (**B**) *n* = 9 (CTRL), *n* = 8 (VPA); (**C**) *n* = 5 (CTRL), *n* = 4 (VPA); (**D**) *n* = 5 (CTRL), *n* = 4 (VPA); and *n* = (3–5) in the cerebral cortex: (**E**) *n* = 3 (CTRL), *n* = 5 (VPA); (**F**) *n* = 3 (CTRL), *n* = 5 (VPA); (**G**) *n* = 4 (CTRL), *n* = 5 (VPA); (**H**) *n* = 4 (CTRL), *n* = 5 (VPA).*** *p* < 0.001, **** *p* < 0.0001 vs. control.

To study the possible involvement of mitogen-activated protein kinases: ERK1 and ERK2 in VPA-evoked Tau phosphorylation we analysed the level of phosphorylated p44MAPK (p-ERK1) at (Thr202/Tyr204), p42MAPK (p-ERK2) at (Thr185/Tyr187) as well as the level of total p44/42MAPK (ERK1/2). Our study revealed that exposure to VPA during embryonic development had no effect on the level of p-ERK1/2 in the hippocampus (Figure 11A,B). In turn, in the cerebral cortex, the exposure to VPA evoked a significant increase in the level of both p-ERK1 and p-ERK2 protein (508% increase in p-ERK1, *p* < 0.0001 and 124% increase in p-ERK2, *p* = 0.0008), compared to the respective control groups (Figure 11E,F). Analysis of the expression of total ERK1/2 revealed the lack of changes in protein level in the hippocampus and cerebral cortex of animals exposed to VPA (Figure 11C,D,G,H). The above data indicate stimulation of ERK1/2 activity exclusively in the cerebral cortex of VPA offspring.

**Figure 12:**
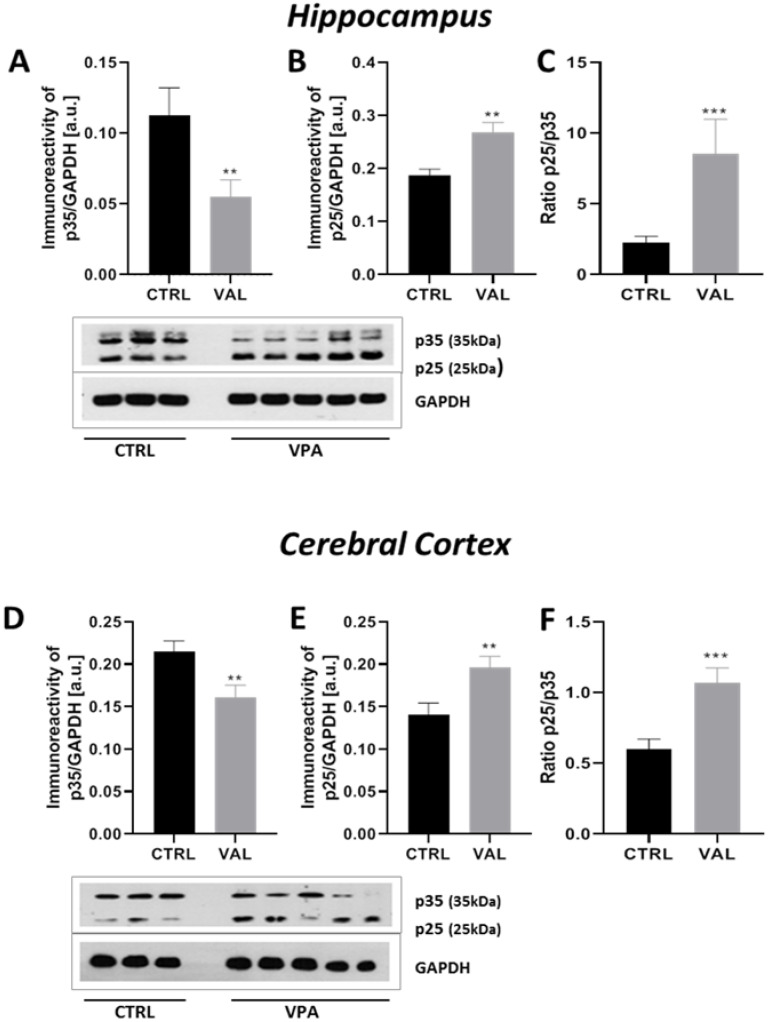
The effect of prenatal exposure to VPA on the calpain-dependent activation of CDK5 kinase in the rat brain. Immunoreactivity of p35 and its degradation product p25 were determined using Western blot analysis. Densitometric analysis and representative pictures of p35 and p25 in the hippocampus (**A**,**B**) and cerebral cortex (**D**,**E**) were shown. Results were normalized to GAPDH levels. Additionally, the ratio of p25/p35 in the hippocampus (**C**) and cerebral cortex (**F**) were measured. Data represent the means ± S.E.M. from *n* = (11–14) independent experiments in both the hippocampus and cerebral cortex: (**A**) *n* = 11 (CTRL), *n* = 14 (VPA); (**B**) *n* = 11 (CTRL), *n* = 14 (VPA); (**C**) *n* = 11 (CTRL), *n* = 14 (VPA); (**D**) *n* = 11 (CTRL), *n* = 14 (VPA); (**E**) *n* = 11 (CTRL), *n* = 14 (VPA); (**F**) *n* = 11 (CTRL), *n* = 14 (VPA). ** *p* < 0.01, *** *p* < 0.001, vs. control.

**Figure 13:**
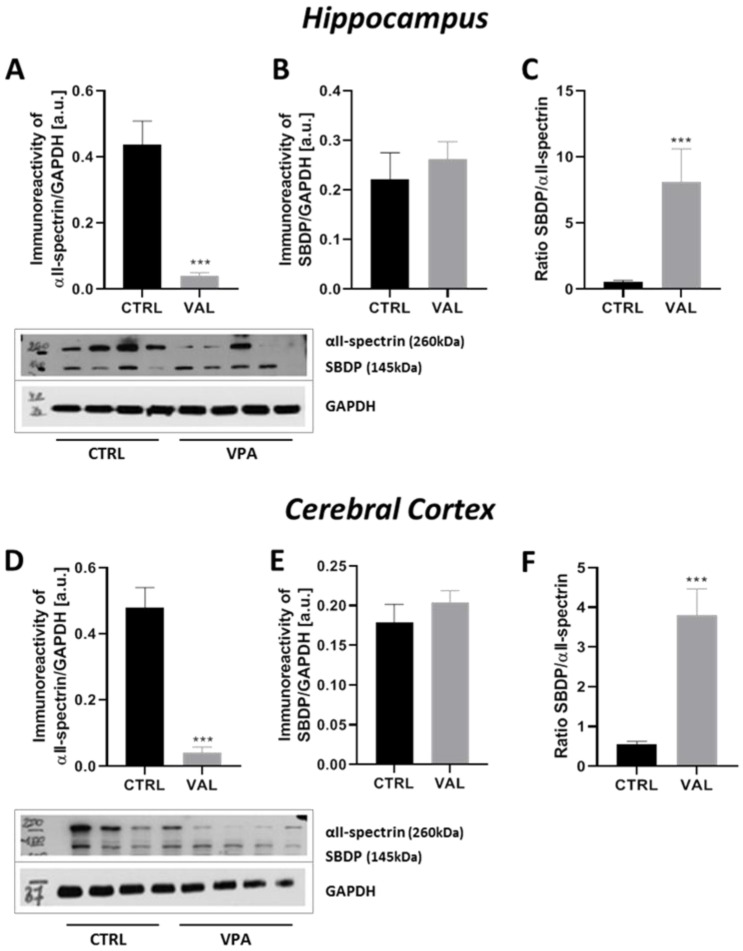
The effect of prenatal exposure to VPA on the calpain-dependent cleavage αII-spectrin and spectrin breakdown product (SBDP) generation in the rat brain. Immunoreactivity of αII-spectrin and its degradation product SBDP (αII-spectrin breakdown product, 145 kDa) were determined using Western blot analysis. Densitometric analysis and representative pictures of αII-spectrin and SBDP in the hippocampus (**A**,**B**) and cerebral cortex (**D**,**E**) were shown. Results were normalized to GAPDH levels. Additionally, the ratio of SBDP/αII-spectrin in the hippocampus (**C**) and cerebral cortex (**F**) were measured. Data represent the means ± S.E.M. from *n* = (7–9) independent experiments in the hippocampus: (**A**) *n* = 8 (CTRL), *n* = 8 (VPA); (**B**) *n* = 7 (CTRL), *n* = 9 (VPA); (**C**) *n* = 7 (CTRL), *n* = 7 (VPA); and *n* = (10–11) in the cerebral cortex: (**D**) *n* = 11 (CTRL), *n* = 10 (VPA); (**E**) *n* = 11 (CTRL), *n* = 10 (VPA); (**F**) *n* = 11 (CTRL), *n* = 10 (VPA).*** *p* < 0.001, vs. control.

The activity of the next major Tau kinase, CDK5 in neuronal cells is dependent on association with its neuron-specific activator p35. Calpain-dependent proteolytic cleavage of p35 to p25 causes prolonged overactivation and mislocalization of CDK5. Therefore, here we analysed the p35 level and its cleavage to p25. As presented in Figure 12A,D, exposure to VPA during foetal life induced a significant decrease in the level of p35 protein in both investigated brain structures (by about 52% in the hippocampus, *p* = 0.010 and by about 25% in the cerebral cortex, *p* = 0.0087) along with rising in the level of p25 protein in both the hippocampus by about 43% (*p* = 0.0025) and in the cerebral cortex by about 40% (*p* = 0.0057) of VPA offspring (Figure 12B,E). Moreover, the ratio of p25/p35 protein was significantly raised in both analysed structures of VPA-exposed brains and was approximately 260% in the hippocampus (*p* = 0.0007) and 66% in the cerebral cortex (*p* = 0.0004) (Figure 12C,F). Thus, prenatal exposure to VPA significantly stimulated calpain-dependent cleavage of p35 to p25 in the hippocampus and cerebral cortex, indicating activation of CDK5 kinase in these brain structures. To confirm the stimulating properties of VPA on the calpain-dependent activation of the CDK5/p25 complex we also analysed the level of 145 kDa spectrin breakdown product (SBDP) protein, which is generated in consequence to the calpain-catalysed breakdown of actin-crosslinking protein, αII-spectrin. As shown in Figure 13, prenatal exposure to VPA evoked significantly and drastic depletion in the level of αII-spectrin in both the hippocampus (by about 91%, *p* < 0.0001) and in the cerebral cortex (by about 86%, *p* < 0.0001) (Figure 13A,D). However, the protein level of SBDP was unchanged (Figure 13B,E). Despite the lack of significant changes in the protein level of SBDP, the ratio of SBDP/αII-spectrin was significantly raised in both analysed brain structures of rats exposed prenatally to VPA (in the hippocampus this ratio was by about 14 times greater than in control, *p* = 0.0006; in the cerebral cortex by about 10 times, *p* < 0.0001) (Figure 13C,F). Additionally it is noteworthy that αII-spectrin, just as Tau and α/β-tubulin, is one of the major cytoskeletal proteins and its depletion may lead to cytoskeletal damage just like a prolonged activation of calpains.

### 2.4. Prenatal Exposure to VPA Up-Regulated the mTOR Signalling 

**Figure 14:**
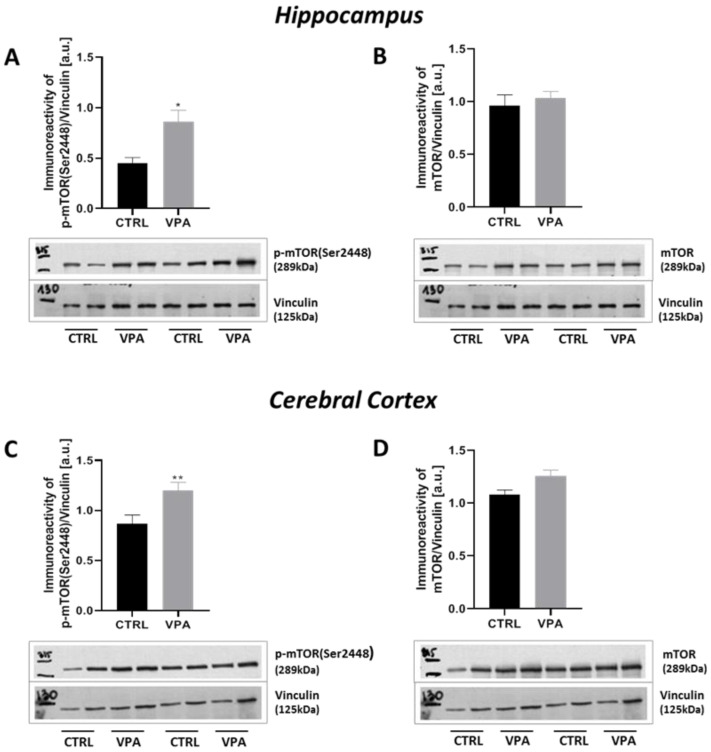
The effect of prenatal exposure to VPA on the mTOR in the rat brain. Immunoreactivity of phospho-mTOR(Ser2448) and total mTOR were determined using Western blot analysis. Densitometric analysis and representative pictures of phospho-mTOR(Ser2448) and mTOR in the hippocampus (**A**,**B**) and cerebral cortex (**C**,**D**) were shown. Results were normalized to Vinculin levels. Data represent the means ± S.E.M. from *n* = (4) independent experiments in the hippocampus: (**A**) *n* = 4 (CTRL), *n* = 4 (VPA); (**B**) *n* = 4 (CTRL), *n* = 4 (VPA); and *n* = (5) in the cerebral cortex: (**C**) *n* = 5 (CTRL), *n* = 5 (VPA); (**D**) *n* = 5 (CTRL), *n* = 5 (VPA). * *p* < 0.5, ** *p* < 0.01, vs. control.

**Figure 15:**
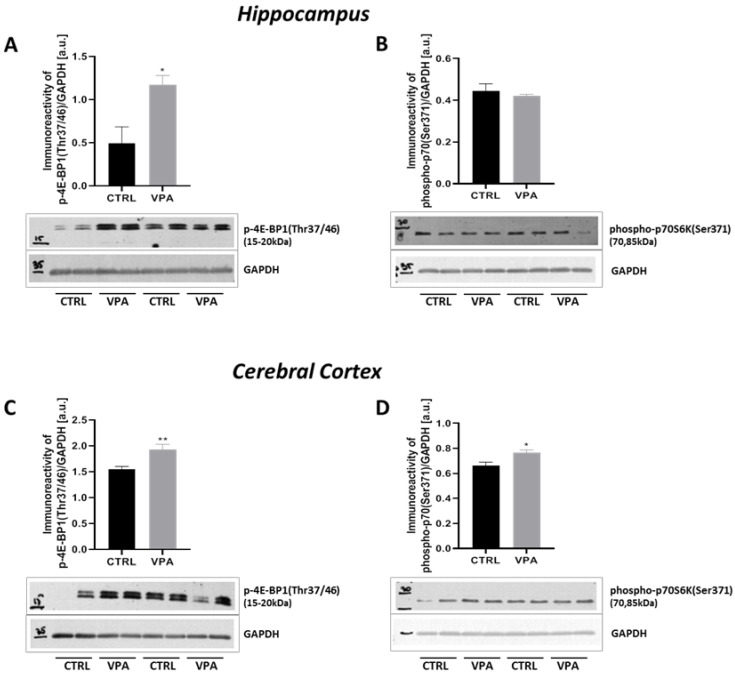
The effect of prenatal exposure to VPA on the mTOR downstream effectors: 4E-BP1 and p70S6K in the rat brain. Immunoreactivity of p-4E-BP1(Thr37/46) and phosho-p70S6K(Ser371) were determined using Western blot analysis. Representative blots and densitometric analysis of p-4E-BP1(Thr37/46) and p70S6K phosphorylated at (Ser371) in the hippocampus (**A**,**B**) and cerebral cortex (**C**,**D**) were shown. Results were normalized to GAPDH levels. Data represent the means ± S.E.M. from *n* = (3–4) independent experiments in the hippocampus: (**A**) *n* = 3 (CTRL), *n* = 4 (VPA); (**B**) *n* = 4 (CTRL), *n* = 4 (VPA); and *n* = (4–8) in the cerebral cortex: (**C**) *n* = 7 (CTRL), *n* = 8 (VPA); (**D**) *n* = 4 (CTRL), *n* = 4 (VPA). * *p* < 0.5, ** *p* < 0.01, vs. control.

Since deregulation of the mTOR is linked to several neurological diseases, including ASD [48,49,50,51] as well as its activation leads to Tau dyshomeostasis by regulation Tau synthesis, phosphorylation, and degradation [52,53], we investigated whether prenatal exposure to VPA affects mTOR activity. First, we examined the level of total mTOR and its phosphorylation on serine 2448 (p-mTOR(Ser2448)), as an established marker of mTORC1 activation. As shown in Figure 14A, the immunoreactivity of the p-mTOR(Ser2448) was markedly increased by about 94% (*p* = 0.0155) in the hippocampus of VPA adolescent offspring. In addition, no difference was found in the total amount of mTOR (Figure 14B). Similarly to the hippocampus, in the cerebral cortex the prenatal exposure to VPA evoked a significant (*p* = 0.0043) increase in the level of p-mTOR(Ser2448) by about 39% without changes in the level of total mTOR (Figure 14C,D). Active mTOR transmits a positive signal to its downstream effectors such as p70S6K and participates in the phosphorylation and inactivation of the eIF4E inhibitor, eukaryotic translation initiation factor 4E-binding protein 1 (4E-BP1). Therefore, to confirm the stimulatory effect of VPA on the mTOR activity we examined additionally the immunoreactivity of p70S6K phosphorylated at (Ser371) residue as well as the level of 4E-BP1 phosphorylated at (Thr37/Thr46). Using Western blot analysis, we found a significant increase in the phosphorylation of 4E-BP1 on (Thr37/46) in both the hippocampus (137% increase, *p* = 0.0221) and cerebral cortex (25% increase, *p* = 0.0084) of rat offspring prenatally exposed to VPA, compared to the respective control groups (Figure 15A,C). Moreover, we revealed that exposure to VPA during embryonic development significantly elevated the level of phospho-p70S6K(Ser371) by about 15% (*p* = 0.0253) exclusively in the cerebral cortex (Figure 15B,D).

### 2.5. Prenatal Exposure to VPA Induced Histopathological Changes in the Neurons of Rat Brain

**Figure 16:**
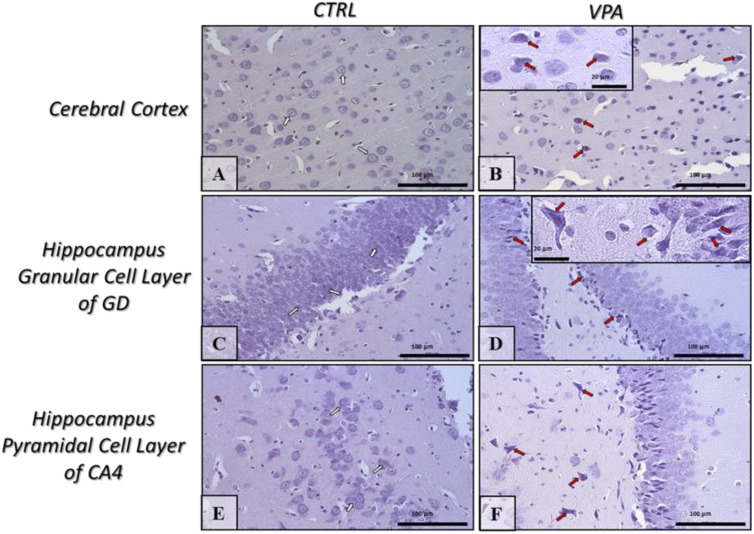
The effect of prenatal exposure to VPA on the histopathological changes in the neurons of the rat brain. Representative microphotography showing the histological structure of chosen brain regions (hippocampus and cerebral cortex) in control (**A**,**C**,**E**) and VPA-exposed rats (**B**,**D**,**F**). H&E staining. Scale bar: 100 µm and 20 µm in the insertion of B and D (objective magnification ×40, ×100, respectively). White arrows—normal nerve cells, red arrows—neurons that shown chromatolysis (acidophilic degradation of Nissl bodies and pyknotic nucleus). Representative pictures from *n* = 11 (CTRL) and *n* = 9 (VPA) independent experiments in both the hippocampus and cerebral cortex are presented.

The changes in the level and phosphorylation of cytoskeletal proteins observed in the rat brain after prenatal exposure to VPA encouraged us to investigate the possible effects of this compound on the structure of nerve cells. The histological analysis of neurons in the hippocampus and in the cerebral cortex of control rats indicated structurally unchanged nerve cells (normal structure of neurons with basophilic cytoplasm in perikaryon, well-defined Nissl bodies, the nucleus in central position) (Figure 16A,C,E, white arrows). However, the images of nerve cells in rats prenatally exposed to VPA revealed pathological changes in the structure of neurons in all examined brain regions. In the brain of rats prenatally subjected to VPA, we observed the pathologic phenotype (chromatolysis) in neurons, that was manifested as eosinophilic cytoplasm in perikaryon, acidophilic degradation of Nissl bodies, and presence of pyknotic nucleus located eccentrically near to the cell membrane (Figure 16B,D,F, red arrows). All these observations may suggest that the cytoskeletal network is negatively affected by VPA.

## 3. Discussion

Our previous study revealed long-term pathological changes in the synaptic ultrastructure in the aftermath of prenatal exposure to VPA that could contribute to the impairment of neuronal function and behavioural abnormalities typical for ASD [14]. 

A growing body of evidence suggest the linkage of a synaptic pathology with dysfunctional MT cytoskeleton. Deregulation of the MT cytoskeletal network is considered to be a common insult during the pathogenesis of many neurodevelopmental diseases including ASD, intellectual disabilities (ID), polymicrogyria, and schizophrenia. This concept is supported by evidence from clinical and animal models studies, which have revealed abnormalities in dendrites, axons, and in the organization of cytoskeleton as well as numerous mutations or changes in the expression of genes encoding tubulins, MT-associated proteins (MAPs), or additional MT regulatory factors [12,21,22,23,24,47,54]. However, changes in the level of key proteins responsible for the MT formation as well as maintenance of their proper structure, function and dynamics were not investigated. 

In the present study, we provide evidence that in the VPA-induced rodent model of autism comes defects in MT assembly and properties of the MT cytoskeleton. The current studies are the first, where we showed a significant decrease in the level of α/β-tubulin together with excessive accumulation of Tau protein in the brain of adolescent rat offspring prenatally exposed to VPA. We also revealed a brain region-dependent Tau protein hyperphosphorylation that was accompanied by deregulation of activity of some Tau kinases. 

The MTs system together with MAPs is important in the regulation of basic neurodevelopmental processes such as neurons generation, polarization, migration, and neuronal branching as well as synapse formation and myelination [20,23]. MTs establish synaptic contacts and contribute to the creation of an effective functional connectivity network. The results of some studies also suggest a vital role of MTs in cognitive functions and behaviours, as they are essential in the growth and maintenance of the axon, the development and plasticity of the dendritic spines, and the migration of developing neurons to their destinations [12]. Structural units of MTs are heterodimers of α and β-tubulin that bind to form 13 protofilaments which associate laterally to form a hollow, polar cylinder with a diameter of 25 nm, and highly variable length [25]. Numerous lines of evidence suggest that mutations in tubulin genes impair different MTs functions and are associated with a wide spectrum of neurological disorders collectively known as “tubulinopathies” which primarily causing neurodevelopmental diseases [22,55,56]. It was shown that human missense heterozygous mutations in the 9 α-tubulin and 10 β-tubulin isoforms forming the heterodimers that assemble into MTs causing malformations of cortical development (MCDs) associated with intellectual disability and refractory childhood epilepsy [57]. Additionally, in post-mortem brains from subjects with schizophrenia or bipolar disorder the expression of β-tubulin was decreased in the prefrontal cortex compared to healthy controls [58]. The study using the rodent model of depression revealed a decrease α/β-tubulin expression in the hippocampus [59]. In our study, we observed a significant decrease in the level of α/β-tubulin which may lead to damage of MTs structure and could have deleterious effects on the cytoskeletal network. In opposite to our study, in C58/J mouse model of ASD, the level of α-tubulin remained unchanged in the hippocampus, prefrontal cortex, and cerebellum [17]. The reason for the discrepancy in the results may be due to differences in the experimental model used. 

Observed in our study decrease in the level of α/β-tubulin could be responsible for morphological changes in neurons indicative of chromatolysis, found in VPA-exposed offspring brains. Chromatolysis is the usually early, often sublethal change that occurs in neuronal injury. It is commonly the result of exposure to toxins, ischemia, infections, and interference with cellular metabolism. Chromatolysis, also known as the axon reaction, occurs when the normal aggregation of rough endoplasmic reticulum and associated ribosomes, known as Nissl substance, in the neuronal perikaryon disperse as a response to injury. At the same time, the cytoskeletal network, which frames the neuronal cytosol and supports the nucleus, is negatively affected. This loss of nuclear suspension leads to the nucleus losing its central position, becoming eccentric, lying adjacent to the cell membrane [60,61]. In our study, the image of nerve cells in rats prenatally exposed to VPA revealed pathological phenotype in neurons that were manifested as eosinophilic cytoplasm in perikaryon, acidophilic degradation of Nissl bodies and presence of pyknotic nucleus located eccentrically near to the cell membrane. Consistently to our results, in the study of Sadhya et al., 2012 early prenatal or postnatal exposure to VPA altered histoarchitecture of the cerebellum [62]. Moreover, Hara et al., 2012 demonstrated that exposure to VPA on embryonic day 12.5 significantly reduced the number of Nissl-positive cells in the prefrontal and somatosensory cortices in male mice and suggested that VPA-induced morphological abnormalities in neurons may be involved in the social interaction deficits [63].

Both the function and organization of MTs, especially neuronal MTs, depend on different MT-associated proteins [55]. Tau is one of the major MAPs expressed in the central nervous system, mainly found in the axon of neurons where it promotes MT assembly and bundle formation [64,65]. Tau binds to MTs and is responsible for the stabilization and regulation of the function of the MT-based cytoskeleton [27]. The biological activity of Tau is regulated by phosphorylation/dephosphorylation cycles. Phosphorylation at specific sites detaches Tau from MTs and allows MT depolymerisation, while Tau dephosphorylation enables it to bind and stabilize the MTs [35]. Hyperphosphorylation of Tau depresses its normal biological activity, making this protein more susceptible to aggregation into insoluble inclusions that destabilize MTs and make neurons more vulnerable and prone to degeneration. Although Tau contains approximately 85 potential phosphorylation sites in its longest isoform, phosphorylation at (Ser396) seems to play a pivotal role in Tau function and in particular depolymerizes and destabilizes MTs [33,34,37]. Increased level of p-Tau(Ser396) and formation of intracellular deposits, known as neurofibrillary tangles (NFTs), has been found in the brain of patients with Alzheimer’s disease (AD), in several tauopathies, in various models of Parkinsonism and synucleinopathies, as well as in the result of perinatal exposure to lead (Pb) [36,37,38,66,67]. Additionally, (Ser199/202) and (Ser416) are critical phosphorylation sites of Tau which have been related to Tau pathology. A quantitative in vitro study demonstrated that phosphorylation of Tau at (Ser199/202) is among the critical sites that convert Tau to a toxic-like protein [68]. Also (Ser416) is the AD-related site of Tau phosphorylation which is associated with the weakening of strengthened synapses [69].

Novel and more recent studies revealed changes in Tau protein expression in autistic patients suggest the importance of dyshomeostasis of this protein in the aetiology of ASD [28,41,42]. There are indications that suggest that “Alzheimer’s protein” may turn up as a potential target for autism treatments after that in murine models of ASD a genetic reduction in Tau levels eases the symptoms typical for autism [40]. In the current study, we have shown for the first time that exposure to VPA in utero promotes Tau accumulation as well as a brain region-dependent Tau hyperphosphorylation, which may contribute to MTs destabilization and synaptic endings dysfunction. Excessive Tau phosphorylation results in the loss of its activity and function as well as in a gain of a toxic property whereby sequesters not only normal Tau, but also the other MAPs, leading to disruption of MTs [70]. Our results indicated an increase in Tau phosphorylation at both (Ser396) and (Ser416) sites in the cerebral cortex. However, Baron-Mendoza et al., 2018 showed a decrease in (Ser396) phosphorylation in the brain of the autistic-like mouse strain C58/J [17]. Therefore, we are suggested that changes in Tau phosphorylation state depend on the cause and form of autism. Induced by prenatal exposure to VPA the abnormal Tau phosphorylation could predispose the brain to the aggregation and accumulation of this protein in older individuals and potentially lead to the development of neurodegenerative diseases. It was indicated that more than 10 percent of people diagnosed with autism from age 40 to 60 develop a dementia condition such as AD within 15 years [71]. Moreover, recent research data in a geriatric cohort with mild cognitive impairment or early dementia demonstrate that ASD behaviours may appear de novo of degenerative dementia and autistic behaviours are more prevalent in those with early-onset dementia [72]. The same research group indicated a correlation of autism-like behaviours with increased levels of Tau and neurofibrillary pathology in the frontal lobes at autopsy in subjects with late-life dementia [73]. 

Hyperphosphorylation of Tau may alter its degradation and its truncation by proteases, which can affect total Tau levels [74]. The observed Tau accumulation might be also a consequence of enhanced protein synthesis and/or the inhibition of Tau degradation. The increase in the level of Tau in VPA brains may be the result of the activation of mTOR kinase, which up-regulates protein translation and inhibits the autophagy process [75]. Deregulation of mTOR is linked to several neurological diseases, including ASD [51]. Compelling evidence has suggested that activation of the mTOR signalling cascade enhances Tau pathology by increasing Tau protein level and its phosphorylation [53]. In our study, we indicated mTOR activation in both the cerebral cortex and hippocampus after prenatal exposure to VPA. The active mTOR complex 1 (mTORC1) transmits a positive signal to its downstream effector p70S6K as well as participates in the phosphorylation and thus inactivation of the 4E-BP1. Observed in our study an increase phosphorylation state of both p70S6K and 4E-BP1 may suggest the stimulation of translation initiation of specific mRNA subpopulations, including probably mRNA for Tau protein, which could explain the excessive accumulation of Tau in our experimental conditions. Gene sequence comparison identified that Tau mRNA belongs to the 5′ top mRNA group. Additionally, it has been established that mTOR activation via downstream p70S6K increases the translation of Tau mRNA [52]. Tau mRNA has 5′ toplike structure that is preferentially regulated by the mTORC1-S6K pathway [52]. Our observations agree with studies of Qin et al., 2016, who observed overactivation of mTOR signalling associated with stimulation of S6K and additionally suppression of autophagy process in a rat model of ASD, induced by VPA [76]. Inhibition of PI3K/Akt/mTOR-mediated autophagy in VPA-exposed rat hippocampi reported by Zhang et al. [77] could be an additional factor responsible for the rise in the level of Tau protein. Additionally, the accumulation of Tau might be a consequence of the alterations in its cleavage by proteases [74]. Since the abnormal hyperphosphorylation of Tau makes it resistant to proteolysis by calcium-activated neutral proteases, this is most likely the reason for a several-fold increase in the levels of Tau in AD [70]. Perhaps the same relationship is possible in VPA-exposed brain, which could also explain the increase in Tau levels in our experimental conditions. 

Tau is a substrate for several protein kinases. Among them, GSK-3β, CDK5, and ERK1/2 have been mostly implicated in the abnormal Tau phosphorylation [78,79]. In our experimental conditions, prenatal exposure to VPA induced an increase in Tau phosphorylation at (Ser396) and (Ser416) exclusively in the cerebral cortex of the rat offspring. Additionally, in this brain structure, we observed significant activation of both ERK1/2 and CDK5 kinase, without changes in GSK-3β activity. Moreover, stimulation of p70S6 kinase activity, which is responsible for both the regulation of synthesis and Tau phosphorylation [52,80], was observed also only in the cerebral cortex. All these data suggest the significant role of ERK1/2, CDK5 as well as p70S6 kinase in VPA-evoked Tau phosphorylation in the cerebral cortex of rat offspring and the lack of direct effect of GSK-3β. Similar to our study, lower GSK-3β activity was observed in children with ASD [48]. VPA is able to activate multiple signal transduction pathways including the PI3K/Akt and MAPK/ERK pathway which leads to an increase in the phosphorylation of GSK-3β on (Ser9), thereby inhibit its activity [81]. Therefore, the stimulation of Akt kinase observed in the hippocampus of VPA rats could explain the inhibition of GSK-3β in this brain structure. Similar to our study, an in vitro investigation of VPA-treated SH-SY5Y cells indicated a large increase in Akt activity together with more modest phosphorylation of GSK-3β on (Ser9) [82]. Our observations agree also with studies by Qin et al., 2016, who observed sequential activation of Akt together with GSK-3β inhibition in the rat brain after VPA exposure [76]. In addition, Hu et al., 2011 observed a marked reduction in GSK-3β activity in APP/PS1 mouse brain exposed postnatally to VPA [83]. Additionally, in the study of Mahmood et al., 2018, VPA injection during embryonic development induced Akt activation associated with dendritic spine anomalies [84]. Moreover, in addition to Akt kinase, CDK5 can also indirectly reduce Gsk-3β activity as a Tau kinase [85]. Thus, CDK5 activation could also be responsible for GSK-3β inhibition observed in the hippocampus of VPA rats. In turn, lower GSK-3β activity could lead to TSC2 deregulation and mTOR up-stimulation [86]. Furthermore, TSC2 is directly phosphorylated by Akt at (Thr1462). This phosphorylation inhibits the TSC1/TSC2 complex, a master negative regulator of mTOR [87,88]. Moreover, activated Akt may also directly phosphorylate the mTOR at (Ser2448), leading to its activation [89]. The latest data indicate also that overactivation of the PI3K/Akt/mTOR signalling pathway may be a result of Tau accumulation, after that in murine models of ASD the inhibitory interaction of Tau with phosphatase and tensin homolog (PTEN) (a negative PI3K/Akt regulator) via Tau’s proline-rich domain was discovered [40]. Considering all these molecular mechanisms and the relationships between them, we suggest that stimulation of mTOR along with inhibition of the downstream 4E-BP1 in the hippocampus of VPA-exposed rats could be due to activation of Akt kinase with simultaneous inhibition of GSK-3β. However, the lack of p70S6K activation remains unclear. The unchanged activity of this kinase combined with lack of changes in the activity of ERK1/2 and inhibition of GSK-3β may, in turn, explain the lack of changes in Tau phosphorylation in the hippocampus. Despite CDK5 activation, it appeared to be insufficient to generate phospho-Tau in the hippocampus of VPA-exposed rats.

A different effect of VPA on the Tau kinases activity we observed in the cerebral cortex of young-adult offspring. In this brain structure, in addition to CDK5 activation, we revealed stimulation of ERK1/2 and downstream mTORC1 effector p70S6K that are able to Tau phosphorylation [52,78,90,91]. There are converging preclinical and clinical evidence that suggests that perturbations in the MAPK/ERK signalling pathway are linked to a group of related neurodevelopmental disorders hallmarked by intellectual disability, including autism [48,50,92]. ERKs play a critical role in brain development and synaptic plasticity [93,94] and importantly, they are genetically linked to ASD and other syndromes typified by ID [95,96,97,98]. Our study revealed that exposure to VPA during embryonic development evoked a significant increase in the level of both p-ERK1(Thr202/Tyr204) and p-ERK2(Thr185/Tyr187) without the effect on the total level of ERK1/2, thus indicating activation of these enzymes in the cerebral cortex. The majority of data indicate up-regulation of ERKs in ASD. The increase in both protein expression and activities of ERK1/2 was detected in the frontal cortex of autistic subjects [92]. The higher activity of ERK2 was observed in T cells from children with ASD [48]. Moreover, VPA is able to induce the ERK1/2 activation [81,99,100,101]. Additionally, BTBR mice showed positive p-ERK1/2 immunolabeling levels in the prefrontal cortex that negatively correlated with cognitive function [102]. In addition, the pharmacological inhibition of ERK signalling during a critical period of brain development rescued the molecular, anatomical, and behavioural deficits in the 16p11.2 deletion mice [93]. Activated ERK1/2 may directly phosphorylate Tau protein at a large number of the same sites seen in AD [79,91,103]. In opposition to CDK5, which phosphorylates Tau in (Ser396) residue, the MAP kinase family may phosphorylate Tau at both (Ser396) as well as (Ser416) epitope [69,91,104]. It could explain the observed in the cerebral cortex of VPA rats significantly increase in the level of phospho-Tau phosphorylated at both (Ser396) and (Ser416) as well as suggest a probably a pivotal role of ERK1/2 in the stimulation of Tau phosphorylation in this brain structure. However, we cannot exclude the involvement of CDK5 and other Tau kinases in VPA-induced hyperphosphorylation of Tau in the cerebral cortex. ERK1/2 kinase has a broad range of downstream substrates. For instance, cytosolic ERK1/2, in addition to phosphorylation of cytoskeletal proteins, may also phosphorylate TSC2 and ribosomal S6 kinases (RSK) [89,105,106]. Based on the present data we suggest that ERK1/2, activated in the cerebral cortex of VPA-exposed animals, could phosphorylate and inhibit TSC2, which stimulates mTORC1. Activated ERK1/2 may also directly phosphorylate mTOR at (Ser2448), thus activating mTORC1 signalling pathway [105,106]. In turn, the active mTORC1 could transmit a positive signal to its downstream effector p70S6K, which may be also phosphorylated (Thr421/Ser424) and activated immediately by ERK1/2 [107,108]. p70S6K, a central regulator of protein biosynthesis, involved in the up-regulation of Tau mRNA translation may also phosphorylate Tau [80,109]. The study by An et al., 2003 showed a significant correlation between the levels of phosphorylated/activated p70S6K and the progressive sequence of neurofibrillary changes according to Braak’s criteria [108]. Phosphorylated p70S6K, total Tau and paired helical filaments (PHF)-Tau were significantly increased in the AD brain suggest the involvement of p70S6K in Tau phosphorylation and synthesis. In the study of Pei et al., 2006, the close association of phospho-p70S6K(Thr421/Ser424) with phospho-Tau(Ser262) and (Ser396/404) was demonstrated using the immunoprecipitation technique [80]. All these data suggest that MAPK-ERK1/2/mTORC1 signalling deregulation may be considered as potential triggers of a molecular cascade leading to Tau dyshomeostasis in the cerebral cortex of VPA-exposed offspring. Moreover, the involvement of ERK1/2 as well as p70S6K, in addition to the CDK5 overactivation, in the stimulation of Tau protein phosphorylation is here proposed. 

The limitation of our study, which should be avoided in further research, is that the experiments were performed exclusively on male young-adult offspring. For a long period of time, it was believed that ASD is more frequent in males than in females; however more recent works have questioned this image and strengthened discussion on the topic of sex differences in autism and related disorders. Despite this, previous studies on the VPA model demonstrated significant behavioural and neuroanatomical alterations in male offspring, while female offspring showed only marginal deficits [110,111,112]. Nevertheless, the impact of gender on key VPA-evoked changes presented in this study should be analysed in detail in future research.

## 4. Materials and Methods

### 4.1. Ethical Statement

All experiments conducted with animals were approved by the Local Ethics Committee for Animal Experimentation in Warsaw, Poland (reference number 4/2014, 60/2015, 64/2015, 361/2017 WAW2/083/2018, and WAW2/148/2018), and were carried out in accordance with the EC Council Directive of 24 November 1986 (86/609/EEC), following the ARRIVE guidelines and guidelines published in the NIH Guide for the Care and Use of Laboratory Animals, and the principles presented in the “Guidelines for the Use of Animals in Neuroscience Research” by the Society for Neuroscience. Efforts were made to minimize animal suffering and to reduce the number of animals used. All manipulations were performed gently and quickly to avoid stress-induced alterations.

### 4.2. Animals—In Vivo Model of ASD

Pregnant Wistar rats between 12 and 15 weeks of age and weighing 210–250 g were supplied by the Animal House of the Mossakowski Medical Research Centre, Polish Academy of Sciences (Warsaw, Poland), which operates breeding of small rodents with the SPF standard. The animals were maintained under controlled conditions of temperature and humidity with a 12-h light/dark cycle. Procedures involving animals were carried out in strict accordance with international standards of animal care guidelines, and every effort was made to minimize suffering and the number of animals used. According to the study of Schneider and Przewlocki [113], autism spectrum disorder (ASD) model was induced by a single intraperitoneal injection of VPA at a dose of 450 mg/kg of body weight on gestational day 12.5 (experimental group). Pregnant females from control groups received a single intraperitoneal injection of solvent (sterile 0.9% NaCl). All pregnant dams were allowed free access to food and water and were kept in a room with a controlled temperature under a LD 12/12 regime to give birth. All dams were allowed to give birth and nurture offspring under normal conditions. The day of birth was considered as postnatal day (PND) 1. On PND 7 each litter was equalized (random selection) and the number of pups was limited to 10 (both male and female). Offspring (males and females) stayed with their mothers and were fed by them until PND 22–23, and then rat pups were separated and kept in groups of 3 or 4 in open polycarbonate cages in an enriched environment. To avoid the interference of the hormonal disturbances/changes only males were selected for further experimental procedures. To reduce the risk of litter effect animals from at least 3 litters in each experimental group (random selection) were used for the experiments. Adolescent males were sacrificed at PND 58–59 by decapitation; their brains were quickly removed and dissected into two regions: hippocampus and cerebral cortex, and then placed in liquid nitrogen. The samples were stored at −80 °C for further analysis. The results more than twice the standard deviation away from the means were excluded.

### 4.3. Immunochemical Determination of Protein Levels (Western Blot Analysis) 

Immunochemical analysis of proteins level and phosphorylation status was performed by Western blotting method in standard conditions. Tissue samples were homogenized, mixed with Laemmli buffer, and denatured at 95 °C for 5 min. After standard SDS-PAGE separation, the proteins were “wet”-transferred to a nitrocellulose membranes in standard conditions and used for immunochemical analysis with specific antibodies, followed by chemiluminescent detection. The membranes were washed for 5 min in TBST (Tris-buffered saline with Tween 20 buffer: 100 mM Tris, 140 mM NaCl and 0.1% Tween 20, pH 7.6) and non-specific binding was blocked for 1 h at room temperature (RT) with 2% or 0.5% BSA in TBST or with 5% non-fat milk solution in TBST. Membranes were probed with the following primary antibodies: α/β-tubulin (1:1000), Tau (1:500), pTau(Ser396) (1:250), pTau(Ser199/202) (1:1000), pTau(Ser416) (1:1000), pGsk-3β(Ser9) (1:250), pGsk-3β(Tyr216) (1:250), Gsk-3β (1:1000), pAkt(Ser473) (1:500), Akt (1:750), pp44/p42MAPK(Thr202/Tyr204) (1:1000), p44/p42MAPK (1:1000), p35/p25 (1:1000), αII Spectrin (1:1000), pmTOR(Ser2448) (1:500), mTOR (1:500), p4E-BP1(Thr37/46) (1:250), pp70S6K(Ser371) (1:250) (Table 1). The membranes were washed three times in TBST, incubated for 60 min at RT with appropriate secondary antibodies (1:8000 anti-rabbit or 1:4000 anti-mouse IgG), and washed again 3 × in TBST. Antibodies were detected using chemiluminescent reaction and ECL reagent (Amersham Biosciences, Bath, UK) under standard conditions. After each protein detection, the membranes were stripped (25 mM Glycine-HCl, 1% (*w*/*v*) SDS, pH 2; 30 min at room temperature) and re-probed. As the first, phosphorylated protein was immunodetected, then the total level, and finally, GAPDH or vinculin. Glyceraldehyde 3-phosphate dehydrogenase (GAPDH, 1:50,000) or vinculin (VCL, 1:1000) was analyzed as a loading control. In all experiments, densitometric analysis of immunoblots was performed using normalization to immunoreactivity of GAPDH or vinculin. Densitometric analysis and size-marker based verification was performed with TotalLab software.

### 4.4. Immunohistochemistry Analysis 

The dissected brains were fixed in formalin. Then, the brains were washed with absolute ethanol (3 times within 3 h), absolute ethanol with xylene (1:1) (twice within 1 h), and xylene (3 times within 20 min). Then, following 3 h of saturation of the tissues with liquid paraffin, the samples were embedded in paraffin blocks. Using a microtome (Microm HM340E), 3–5 µm serial sections were cut and placed on polysine histological slides (ThermoFisher Scientific, Waltham, MA, USA, J2800AMNZ). The sections of the brains were deparaffinised in xylene and rehydrated in decreasing concentration of ethanol, and then used for immunohistochemical (IHC) staining. In order to expose the epitopes, the sections were boiled twice in a microwave oven (700 W for 4 and 3 min) in 10 nM citrate buffer (pH 6.0). Once cooled and washed with PBS, the endogenous peroxidase was blocked by 3% solution of perhydrol in methanol, and then the slides were incubated over the night at 4 °C with primary antibodies. To visualize the antigen-antibody complex, a Dako LSAB + System-HRP was used (DakoCytomation, Glostrup, Denmark, K0679), based on the reaction of avidin-biotin-horseradish peroxidase with DAB as a chromogen, according to the included staining procedure instruction. Sections were washed in distilled water and counterstained with hematoxylin. For a negative control, specimens were processed in the absence of primary antibodies. Positive staining was defined microscopically (Leica DM5000B, Wetzlar, Germany) by visual identification of brown pigmentation. Primary antibody list: Tau (Santa Cruz Biotechnology, Dallas, Texas, USA, sc-32274, final dilution 1:500), pTau(Ser416) (Cell Signalling Technology, Danvers, MA, USA, 15013, 1:200), pTau(Ser396) (Cell Signalling, 9632, 1:200), pTau(Ser199/202) (Sigma Aldrich, St. Louis, MO, USA, T6819, 1:1000), α/β-tubulin (Cell Signalling, 2148S, 1:100).

### 4.5. Statistical Analysis of Biochemical and Behavioral Results 

Results were expressed as mean values ± S.E.M. Differences between the means were analysed using Student’s t-test for two groups; differences were considered significant at *p* < 0.05. Normality and equality of group variances were tested by Shapiro-Wilk test. For non-normal distribution of data, the non-parametrical tests, i.e., U-Mann-Whitney test was used. Statistical analysis was performed using Graph-Pad Prism version 8.0 (Graph Pad Software, San Diego, CA, USA).

## 5. Conclusions

**Figure 17:**
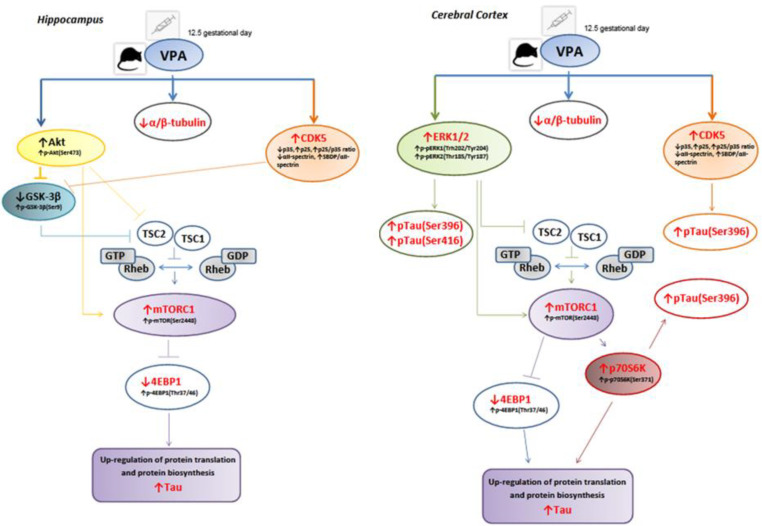
Schematic diagram showing the effect of prenatal exposure to VPA on the pathological changes in the brains of young-adult male offspring. A single i.p. injection of VPA at 12.5 days of pregnancy induced a disturbance in homeostasis of microtubule-associated proteins (significantly decrease in the level of α/β-tubulin together with an excessive rise in total Tau level) in the hippocampus and cerebral cortex of young-adult offspring. Additionally, hyperphosphorylation of Tau protein (significantly increase in the level of pTau(Ser396) and pTau(Ser416)) was observed exclusively in the cerebral cortex of VPA rats. The effect of VPA on the potential molecular mechanisms responsible for Tau dyshomeostasis as well as the Tau-kinases activity was brain structure-specific. In the hippocampus, deregulation of Akt/GSK-3β/mTORC1 pathway and activation of CDK5 kinase have been proposed as potential triggers of a molecular cascade leading to Tau accumulation. In turn, in the cerebral cortex, the involvement of MAPK-ERK1/2/mTORC1 signalling deregulation in Tau dyshomeostasis is suggested. Moreover, in addition to the CDK5 kinase, the involvement of ERK1/2 and p70S6K has been proposed as critical in the stimulation of Tau protein phosphorylation in the cerebral cortex of VPA offspring.

In conclusion, our studies for the first time indicated that maternal exposure to VPA induced a drastic decrease in the level of α/β-tubulin and significant accumulation of microtubule-associated protein Tau in the cerebral cortex and hippocampus of adolescent animals. Moreover, prenatal exposure to VPA led to brain region-specific Tau hyperphosphorylation. Deregulation of Akt/GSK-3β/mTORC1 pathway and activation of CDK5 have been proposed as potential triggers of a molecular cascade leading to Tau accumulation in the hippocampus, whereas the involvement of MAPK-ERK1/2/mTORC1 signalling is suggested in Tau up-regulation in the cerebral cortex. Moreover, ERK1/2 as well as p70S6K, in addition to the CDK5 overactivation, could be involved in Tau hyperphosphorylation in this brain structure (Figure 17, Table 2). It seems that activation of mTOR kinase-dependent signalling plays a pivotal role in Tau protein alterations in both the cerebral cortex and hippocampus of adolescent rat offspring prenatally exposed to VPA. The observed changes in Tau protein together with a significant decrease in α/β-tubulin level may suggest destabilization and thus dysfunction of the MT cytoskeleton network, which in consequence may lead to the disturbances in synaptic structure and function, that is associated with the neuroplasticity alterations in the development of autistic-like behaviours.

## Figures and Tables

**Table 1:** Experimental conditions used to perform the Western blot experiments.

Primary Antibody	Brand/Cat #	Dilution
Rabbit anti-α/β-tubulin	Cell Signalling 2148S	1:1000 5% BSA in TBS-T 0.1%
Mouse anti-Tau	Santa Cruz Biotechnology sc-32274	1:500 5% milk in TBS-T 0.1%
Mouse anti-pTau(Ser396)	Cell Signalling 9632	1:250 TBS-T 0.1%
Rabbit anti-pTau(Ser199/202)	Sigma T6819	1:1000 5% milk in TBS-T 0.1%
Rabbit anti-pTau(Ser416)	Cell Signalling 15013P	1:1000 5% milk in TBS-T 0.1%
Mouse anti-pGsk-3β(Ser9)	Santa Cruz Biotechnology sc-373800	1:250 5% milk in TBS-T 0.1%
Mouse anti-pGsk-3β(Tyr216)	BD Diagnostic 612313	1:250 0.1% BSA in TBS-T 0.1%
Rabbit anti-Gsk-3β	Cell Signalling 9315	1:1000 5% milk in TBS-T 0.1%
Rabbit anti-pAkt(Ser473)	Cell Signalling 4060	1:500 2% BSA in TBS-T 0.1%
Rabbit anti-Akt	Cell Signalling 4691	1:750 2% BSA in TBS-T 0.1%
Mouse anti-pp44/p42MAPK(Thr202/Tyr204)	Cell Signalling 9106	1:1000 TBS-T 0.1%
Mouse anti-p44/p42MAPK(Thr202/Tyr204)	Cell Signalling 4696	1:1000 5% milk in TBS-T 0.1%
Rabbit anti-p35/p25	Cell Signalling 2680 and Santa Cruz Biotechnology sc-820 (Mix)	1:1000 1% BSA in TBS-T 0.1%
Mouse anti-αII-spectrin	Santa Cruz Biotechnology sc-46696	1:1000 5% milk in TBS-T 0.1%
Rabbit anti-p-mTOR(Ser2448)	Cell Signalling 5536	1:500 TBS-T 0.1%
Rabbit anti-mTOR	Cell Signalling 2983	1:500 TBS-T 0.1%
Rabbit anti-p-4E-BP1(Thr37/46)	Cell Signalling 2855	1:250 TBS-T 0.1%
Rabbit anti-p-p70S6K(Ser371)	Cell Signalling 9208	1:250 TBS-T 0.1%
Rabbit anti-GAPDH	Sigma-Aldrich G9545-200UL	1:50,000 5% milk in TBS-T 0.1%
Rabbit anti-vinculin	Cell Signalling 13901	1:1000 5% milk in TBS-T 0.1%
**Secondary antibody**	**Brand/cat #**	**Dilution**
anti-mouse IgG	GE Healthcare VXA931V	1:4000 5% milk in TBS-T 0.1%
anti-rabbit IgG	Sigma-Aldrich A0545-1ML	1:8000 5% milk in TBS-T 0.1%

**Table 2:** The effect of prenatal exposure to VPA on the changes in neuronal cytoskeletal proteins, the activity of Tau kinases, and deregulation of Akt/mTOR pathway in the rat brain. (↓)—decrease level; (↑)—increase level; (-)—without changes.

Changes in Neuronal, Cytoskeletal Proteins		
	Hippocampus	Cerebral Cortex
α/β-tubulin	↓	↓
MAP-Tau	↑	↑
pTau(Ser396)	-	↑
pTau(Ser199/202)	-	-
pTau(Ser416)	-	↑
αII-spectrin	↓	↓
**Changes in Tau kinases involved in Tau phosphorylation**		
p-GSK-3β(Ser9)	↑	-
p-GSK-3β(Tyr216)	-	-
GSK-3β	-	-
p35	↓	↓
p25	↑	↑
p25/p35	↑	↑
SBDP/αII-spectrin	↑	↑
p-pERK1(Thr202/Tyr204)	-	↑
pERK1	-	-
p-pERK2(Thr185/Tyr187)	-	↑
pERK2	-	-
**Changes in Akt/mTOR pathway involved in regulation of Tau homeostasis**		
p-Akt(Se473)	↑	-
Akt	-	-
p-mTOR(Ser2448)	↑	↑
mTOR	-	-
p-4EBP1(Thr37/46)	↑	↑
p-p70S6K(Ser371)	-	↑

## Data Availability

The data presented in this study are available on request from the corresponding author.

## Abbreviations

ASD	Autism Spectrum Disorder
VPA	Valproic acid
MTs	Microtubules
MAPs	Microtubule-associated proteins
Tau	Tubulin-associated unit
GSK-3β	Glycogen synthase kinase-3β
MAPK	Mitogen-activated protein kinase
CDK5	Cyclin-dependent kinase 5
PKA	cAMP-dependent protein kinase A
PKC	Protein kinase C
NFTs	Neurofibrillary tangles
mTOR	Mechanistic target of rapamycin/mammalian target of rapamycin
MAPK/ERK	Mitogen-activated protein kinase/extracellular signal-regulated kinase
ERK1	Extracellular signal-regulated kinase 1
ERK2	Extracellular signal-regulated kinase 2
SBDP	αII-spectrin breakdown product
mTORC1	mTOR complex 1/Mechanistic target of rapamycin complex 1
p70S6K	p70 ribosomal protein S6 kinase
4E-BP1	Eukaryotic translation initiation factor 4E-binding protein 1
eIF4E	Eukaryotic translation initiation factor 4E
ID	Intellectual disabilities
Pb	Lead
AD	Alzheimer’s disease
PDPKs	Proline-dependent protein kinases
LTD	Long-term depression
PI3K/Akt	Phosphatidylinositol 3-kinase/protein kinase B
RheB1	GTP-bound levels of small GTPase Ras homolog enriched in brain
TSC2	Tuberous sclerosis complex 2
PTEN	Phosphatase and tensin homolog
AGC	Kinase group of kinases related to PKA PKG and PKC
PHF	Paired helical filaments
PND	Postnatal day
